# Mad River Valley Dental Society

**Published:** 1872-10

**Authors:** D. Gale French


### Mad River Valley Dental Society.

The Mad River Valley Dental Society held its regular semi-annual meeting in the parlors of the Lagonda House, Springfield, Ohio, September 24th, 1872, with President Watt in the chair. The meeting was opened with prayer by Dr. J. H. Paine. The minutes of the last meeting were read and approved. Dayton, Ohio, was then selected as the permanent place for holding the regular meetings. Dr. Blount presented Dr. F. C. Runyon's name for membership, who was unanimously elected. On presentation of Saml. E. Goe's name, by Dr. J. H. Paine, he was elected honorary member.

The bill of the Cor. Secy. to the amount of $3. 90 was allowed and ordered paid. On motion of Dr. Paine, a vote of thanks was tendered the Cor. Secy. for the programmes furnished the society. The bill of the Rec. Secy. to the amount of $4. 38 was allowed and ordered paid. The meeting then adjourned until 2 P. M.

AFTERNOON SESSION.

The meeting was called to order promptly at 2 o'clock. The minutes of the morning session were read and approved, when Dr. A. A. Blount, favored the society with a retiring Presidential address which was to have been read at the last meeting. The paper was ordered read and filed. When the funds of the association shall have accumulated to the sum of twenty-five dollars over and above the current expenses, the same are to be forwarded to the Ohio Dental College, the society to be credited with these donations until one share of stock can be bought.

Dr. J. H. Paine read an essay on aching teeth. Considerable discussion followed on the paper, which was received and filed. Dr. A. A. Blount presented Dr. H. W. Morey's name for membership, who was duly elected. The subject of aching teeth and exposed nerves was introduced by Dr. Rawson, who discussed at some length the best methods of capping nerves; has found willow wood (when nicely fitted) to be the best, inasmuch as it is a perfect non-conductor.

Dr. French thought better success would follow by the use of "Hills' stopping," with a covering over it of Guillois, cement, whereby you get a perfect adaptation, much better than is possible with Dr. R's "willow wood," with nearly a non-conductor, and the cement makes a more solid foundation. Dr. Blount thinks oxychloride of zinc impracticable, and would never expect to save a nerve alive when it is applied. Dr. Tizzard differed somewhat from Dr. Blount, and has been eminently successful in many cases. Dr. McGinnis thought he had failed more times than succeeded in capping nerves. Dr. Rawson gave some advice, from his own experience. The subject was here delayed to hear an essay on periostitis, by Dr. J. A. Stipp, which was finely written. The paper was received and ordered filed.

Exposed nerves was again commenced by Dr. Geo. Watt, who speaks with a wonderfully telling effect upon his hearers, who listened with marked attention, and if his advice is followed much good must ensue. The best mode of separating teeth preparatory to filling proximal cavities was the next subject discussed, Dr. Blount taking the lead, who does not favor heroic wedging; likes cotton better than rubber; thinks rubber too severe. Dr. Rawson does not favor this heroic wedging; he files the teeth apart, files freely. Dr. McGinnis separates with wooden wedges. Dr. French prefers the use of rubber instead of any thing else; thinks it no more severe than cotton or wood; he commences with a thin piece, and then increases gradually until sufficient space is obtained.

Dr. Watt was glad the society denounced heroic wedging, and was proud of its members for it; he always believes in the mildest treatment possible at all times, and under all circumstances; his idea is pressure and rest. Drs. Harlan and Morey were of about the same opinion as Dr. Watt. The meeting adjourned until 7½ P. M.

EVENING SESSION.

The President called the meeting to order promptly at 7½ o'clock. The minutes of the afternoon session were read and approved. Dr. French was then called on to read an essay on "Neatness," which, after being received, was ordered filed. The addition of Dr. J. Taft and Dr. A. Berry to the executive committee was ordered, and the essays given into their charge for further disposition. Dr. J. H. Paine then presented the society with his method of taking an impression with plaster and the "bite" at the same time with the same. But we think the operation too much complicated for recommendation. The best method of arresting *hemorrhage* was discussed by Dr. Watt and others. The chair then appointed, as essayists for the next meeting, Dr. J. H. Paine, S. B. Tizzard, S. H. Harlan A. Berry and F. C. Runyon. The fourth subject was then discussed with considerable spirit by Drs. Paine, Rawson Watt and others. Dr. J. H. Paine thinks that "*celluloid*" is a failure; thinks Dr. Watts, metal far superior, Dr. Rawson argued strongly for rubber; *he* considers it better for gencral use than anything as yet introduced to the profession; says it is *impossible* for dentists to do away (altogether) with its use, and that it required as much time to work it as *gold* or *continuous gum*. Dr. Berry thought it a mistake to say it required as much time as gold, unless one were to sit and look at it. Dr. B. has not much *love* for the rubberr and less for "*Josiah*." Dr. French thought it absurd to say we are compelled to use rubber in any case, and thought the rapid absorption of the boney tissue sufficient to *compel* the use of metal plates. Drs. Berry and Rawson said that there would be just as much absorption with the metal as the rubber. To which Dr. F. demurred, saying that they were the first men (to his knowledge) who ever made such a statement. Dr. Watt said he thought they must be simply mistaken in their observations, for until the laws of nature were changed it would be impossible for absorption to take as fast or as much under a plate that is a conductor of heat and cold as under a non-conductor. The society then adjourned, to meet at Dayton, Ohio, on Tuesday, the first day of April, 1873.

D. GALE FRENCH, Rec. Secy. and Treas.

